# Pakistan’s transgender health disparities—a threat to HPV elimination?

**DOI:** 10.1016/j.lansea.2024.100351

**Published:** 2024-01-16

**Authors:** Usman Ayub Awan, Aamer Ali Khattak, Qian Bai, Suliman Khan

**Affiliations:** a Medical Research Center, The Second Affiliated Hospital of Zhengzhou University, Zhengzhou, China; b Department of Medical Laboratory Technology, The University of Haripur, Haripur, Khyber Pakhtunkhwa, Pakistan; c Division of Epidemiology, Vanderbilt University Medical Center, Nashville, TN, USA; d Department of Anesthesiology, The Second Affiliated Hospital of Zhengzhou University, China

We read with interest the Comment^1^ in *The Lancet Regional Health—Southeast Asia* urging inclusive menstrual health programs for gender-diverse people, including trans and non-binary people, who suffer stigma in healthcare settings. In parallel, we are deeply concerned that screening transgender individuals for human papillomavirus (HPV) is largely neglected in Pakistan, the fifth most populous country in the world with 239.4 million people.^2^ Herein transgender people, known as *hijras*, have long experienced institutional discrimination, assault, and marginalisation. Despite legal recognition in 2010, they confront health inequities and startlingly limited healthcare access, which increases their risk of sexually transmitted infections (STIs). For instance, transgender sex workers (TSWs) are eight times more likely to get HIV than their cisgender counterparts, accounting for 17% of the HIV population in Pakistan.^3^^,^^4^ There was a spike in new HIV cases (n = 8262) between January and September 2022—with 496 cases being reported from Islamabad, the Pakistan’s capital.^5^ TSWs who use drugs actively engage in different sexual activities. A cross-sectional study in Pakistan by Melesse and colleagues showed that 71% of TSWs had sex with other people who use drugs, and 33.7% did not use condoms.^6^ Since HPV is mostly transmitted through sexual contact, sex education among individuals of this community is important.

According to Advisory Committee on Immunization Practices (ACIP)—vaccinating all sex workers (age ≤ 26 years) is recommended considering the higher likelihood of anal cancer.^7^ However, Pakistan lacks a central repository to monitor the incidence of STIs and has no immediate plans to launch a nationwide HPV vaccination campaign targeting heterosexual or Lesbian, Gay, Bisexuals, Transgender, and Queer (LGBTQ+) communities.^8^ In contrast, some Asian nations have already undertaken countrywide HPV vaccination initiatives, including Maldives (in 2019), Thailand (in 2017), Sri Lanka (in 2017), Bhutan (in 2010), and both India and Bangladesh have launched various pilot projects.^9^ Intriguingly, a cross-sectional study in Karachi by Ejaz and colleagues showed that the widespread HPV-subtypes (high-risk: 16, 18, 31, 33, 35, 45, 52, 58, 59, 56, and low-risk: 6/11) among men who have sex with men (MSM) may be addressed by the currently available nonavalent (93.3%) and quadrivalent (84%) vaccines.^10^

Health officials must analyse factors that might hinder or promote vaccination, since transgender people are confronted by health inequities and societal uncertainty. Since 2015, 91 fatalities and more than 2000 incidences of violence against transgender women have been reported in the province of Khyber Pakhtunkhwa.^11^ According to the Trans Murder Monitoring project—a notable rise in the average number of transgender people homicides in Pakistan, from 6 in 2018 to 11 in 2019–2021. Whereas, in 2022, the figure rose to 14—far higher than its neighbours: India with 6, and one each in China, Thailand, and Iraq.^12^ With this targeted stigmatisation and violence, how can awareness campaigns succeed?

Pakistan—is an Islamic country where the norms and values determining everyday life are infused with religious beliefs and sex education is often discouraged.^13^ Open homosexuality is a societal taboo in Pakistan, resulting in social humiliation, estrangement from intimate relationships, and legal prosecution in severe cases.^10^ As a result, it discourages sex workers from getting identification tags and healthcare checkups. Anal cancer is mainly caused by high-risk HPV types, particularly subtype-16, resulting in 80.7% of persistent infections.^14^ Ejaz and colleagues^10^ reported an overall 65% prevalence of HPV in Karachi (predominant subtypes; HPV16 [35.1%]; 18 [23.2%]; 35 [21.1%]; 59 [10.8%]; 58 [8.2%]; 56 [7.7%]; 31 [7.2%]; 45 [7.2%]; 33 [6.7%]; and 52 [6.7%]) among MSM and the TSWs (HIV-positive 87% vs. HIV-negative 48%; *χ*2 p ≤ 0.001).

Alarmingly, MSM and TSWs put their lives at risk to serve 3–5 customers per night for US$0.7–US$1.03 (or more for unprotected sex); hence, financial constraints hinder condom use.^15^ Worse, TSWs in Karachi lacked an adequate understanding of STIs (particularly HPV) and had limited self-risk perception about HPV infection.^8^ First, we strongly recommend a comprehensive cancer database to monitor exact numbers and spatial distribution to enhance access to these vulnerable patients. Second, legal and policy reform, community-led and anti-violence strategies must be implemented to enhance key populations’ health and human rights and access to HIV, STI, and other diagnostic services. Peer outreach and other peer-based services (awareness-raising, drop-in centres, capacity-building, and resource mobilisation) could address the stigmatisation and structural barriers impacting transgender health and rights in Pakistan. Third, Pakistan could learn from Thailand’s 100% Condom Usage Program (CUP)^16^—a clear and enforced policy, multi-sectoral collaboration, training and capacity building, monitoring and evaluation, and sexual awareness for sex workers. Finally, the government should set up large-scale free anal pap-smear screening units and HPV immunisation programs in safe and accessible locations in collaboration with private organisations already working for TSWs and MSM communities. Transgender health is important, let’s eliminate HPV together.

## Contributors

UAA and AAK conceived, designed the study, and interpreted the data. UAA and QB were involved in writing the manuscript’s first draft. SK supervised and did the final correction of the manuscript. All authors approved the final version of the manuscript.

## Declaration of interests

Authors declare no conflicts of interest.

## References

[1] Babbar K., Martin J., Varanasi P., Avendaño I. (2023). Inclusion means everyone: standing up for transgender and non-binary individuals who menstruate worldwide. Lancet Reg Health Southeast Asia.

[2] World Population Review Pakistan population 2023. https://worldpopulationreview.com/countries/pakistan-population.

[3] Ming L.C., Hadi M.A., Khan T.M. (2016). Transgender health in India and Pakistan. Lancet.

[4] Singh S., Ambrosio M., Semini I. (2014). Revitalizing the HIV response in Pakistan: a systematic review and policy implications. Int J Drug Pol.

[5] Samarasekera U. (2022). Pakistan's growing HIV epidemic. Lancet.

[6] Melesse D.Y., Shafer L.A., Shaw S.Y. (2016). Heterogeneity among sex workers in overlapping HIV risk interactions with people who inject drugs: a cross-sectional study from 8 major cities in Pakistan. Medicine.

[7] Markowitz L.E., Dunne E.F., Saraiya M. (2014). Human papillomavirus vaccination: recommendations of the advisory committee on immunization Practices (ACIP). MMWR Recomm Rep.

[8] Ejaz M., Ekström A.M., Ahmed A. (2022). Human papillomavirus associated prevention: knowledge, attitudes, and perceived risks among men who have sex with men and transgender women in Pakistan: a qualitative study. BMC Publ Health.

[9] The Lancet O. (2022). HPV vaccination in south Asia: new progress, old challenges. Lancet Oncol.

[10] Ejaz M., Andersson S., Batool S., Ali T., Ekström A.M. (2021). Anal human papillomavirus infection among men who have sex with men and transgender women living with and without HIV in Pakistan: findings from a cross-sectional study. BMJ Open.

[11] The Guardian Pakistan's transgender women protest against rising tide of violence 2021. https://www.theguardian.com/global-development/2022/apr/01/pakistan-transgender-women-protest-against-rising-tide-of-violence.

[12] Balzer Carsten, Transgender Europe (TGEU) Trans murder Monitoring 2023. https://transrespect.org/en/map/trans-murder-monitoring/?submap=tmm_2021.

[13] Awan U.A., Khattak A.A. (2022). Has Pakistan failed to roll back HPV?. Lancet Oncol.

[14] De Martel C., Ferlay J., Franceschi S. (2012). Global burden of cancers attributable to infections in 2008: a review and synthetic analysis. Lancet Oncol.

[15] Rajabali A., Khan S., Warraich H.J., Khanani M.R., Ali S.H. (2008). HIV and homosexuality in Pakistan. Lancet Infect Dis.

[16] Rojanapithayakorn W. (2006). The 100% condom use programme in Asia. Reprod Health Matters.

